# Propensity score matching/reweighting analysis comparing intravenous golimumab to infliximab for ankylosing spondylitis using data from the GO-ALIVE and ASSERT trials

**DOI:** 10.1007/s10067-020-05051-1

**Published:** 2020-05-04

**Authors:** L. S. Gensler, S. D. Chakravarty, Chris Cameron, S. Peterson, P. Spin, S. Kafka, S. Nair, A. Deodhar

**Affiliations:** 1 Department of Medicine/Rheumatology, University of California, San Francisco, 400 Parnassus Ave, Box 0326, San Francisco, CA 94143-0326 USA; 2 Janssen Scientific Affairs, LLC, Horsham, PA USA; 3 Drexel University College of Medicine, Philadelphia, PA USA; 4 EVERSANA™, Burlington, Ontario Canada; 5 EVERSANA™, 275 Charlotte St. Suite 207, Sydney, Nova Scotia B1P 1C6 Canada; 6 Janssen Global Services, LLC, Horsham, PA USA; 7 Janssen Pharmaceutica NV, Turnhoutseweg 30, 2340 Beerse, Belgium; 8 Oregon Health & Science University, Portland, OR USA

**Keywords:** Active ankylosing spondylitis, Biologic, Clinical trail and efficacy, Disease activity, Infliximab, Intravenous golimumab, Propensity score, Radiographic axial spondyloarthritis

## Abstract

**Objective:**

To compare the relative efficacy of intravenous golimumab (GOL IV) and infliximab (IFX) for active ankylosing spondylitis (AS).

**Methods:**

Propensity score (PS) methods were used to compare the efficacy of GOL IV 2 mg/kg and IFX 5 mg/kg using individual patient data (IPD) from the active arms of the phase 3 GO-ALIVE and ASSERT studies. Outcomes included the proportion of patients with a ≥ 20% improvement in the Assessment of Spondyloarthritis International Society Criteria (ASAS20), change from baseline in Bath Ankylosing Spondylitis Functional Index (BASFI) score, and change from baseline in C-reactive protein (CRP) levels from weeks 4–52.

**Results:**

Before matching, 105 patients were treated with GOL IV and 201 patients were treated with IFX. After matching on all covariates, 118 patients were included in the ASAS20 analysis, 96 in the BASFI analysis, and 160 in the CRP analysis. After matching, GOL IV showed significantly greater improvement in ASAS20 response than IFX for weeks 28–44 (e.g., OR = 9.05 [95% CI 1.62–50.4] at week 44) and was comparable in change from baseline in BASFI scores and CRP levels to IFX at all time points. Results were robust for inclusion of different sets of covariates in scenario analyses.

**Conclusions:**

This is the first analysis of its kind to leverage clinical trial data to compare two biologics using PS methods in the treatment of active AS. Overall, GOL IV was associated with greater improvement in ASAS20 response than IFX in patients with AS at 28, 36, and 44 weeks of follow-up.

**Table:** 

**Key Points** *• Although intravenous golimumab (GOL IV) and infliximab (IFX) are the only two IV-based tumor necrosis factor (TNF) inhibitors with demonstrated phase 3 clinical efficacy in patients with ankylosing spondylitis (AS), no study has evaluated their comparative efficacy in a head-to-head trial.* *• Propensity score matching was used to derive indirect treatment comparisons of GOL IV and IFX for ≥ 20% in the Assessment of Spondyloarthritis International Society Criteria (ASAS20), change in Bath Ankylosing Spondylitis Functional Index (BASFI), and change in C-reactive protein (CRP) using individual patient data from the GO-ALIVE and ASSERT phase 3 trials.* *• Propensity score matched indirect comparisons showed improved relative efficacy of GOL IV compared to IFX; after matching for up to 16 baseline covariates, GOL IV was associated with significantly greater odds of ASAS20 response at weeks 28, 36, and 44 than IFX as well as equivalent changes from baseline in BASFI and CRP.* *• This novel application of propensity score matching using data from phase 3 trials, the first analysis of its kind in AS, allowed adjustment for important imbalances in prognostic factors between trials to generate estimates of comparative efficacy between GOL IV and IFX in the absence of a head-to-head trial between these treatments.*

**Electronic supplementary material:**

The online version of this article (10.1007/s10067-020-05051-1) contains supplementary material, which is available to authorized users.

## Introduction

Active ankylosing spondylitis (AS) is a chronic, immune-mediated, inflammatory condition that primarily affects the sacroiliac joints and spine, and can cause unacceptable symptoms and signs [1–3]. Patients experience severe back pain, stiffness, and loss of spinal mobility, resulting in substantially reduced quality of life [1, 4–9].

The American College of Rheumatology (ACR), the Spondylitis Association of America (SAA), and the Spondyloarthritis Research and Treatment Network (SPARTAN) guidelines, as well as the Assessment of Spondyloarthritis International Society and European League Against Rheumatism (ASAS-EULAR) guidelines, both recommend first-line treatment with non-steroidal anti-inflammatory drugs (NSAIDs) and physical therapy for active AS [3, 10]. Because this approach is ineffective, partially effective, or intolerable in many patients, biologic therapies that target major effector cytokines involved in the inflammatory processes, including tumor necrosis factor (TNF) and interleukin (IL)-17, have been developed [11–13].

Infliximab (IFX) is a Food and Drug Administration (FDA)-approved treatment for patients with AS [14–17]. The efficacy of IFX is primarily supported by data from a phase 3 clinical trial of patients with AS (the ASSERT study) [14]. Intravenous golimumab (GOL IV) has more recently been approved by the FDA for the treatment of AS [18, 19], based on data from the phase 3 clinical trial of patients with AS (the GO-ALIVE study) [18].

Each of these agents was associated with a significantly greater proportion of patients achieving ≥ 20% improvement in the Assessment of Spondyloarthritis International Society Criteria (ASAS20) response and change in Bath Ankylosing Spondylitis Functional Index (BASFI) scores compared with placebo in the respective trials [14, 18]. In addition, IFX was associated with a significant reduction in C-reactive protein (CRP) levels compared with placebo in the ASSERT study, though this was not evaluated in the GO-ALIVE study. No studies have directly compared GOL IV to IFX (the only two IV-based TNFi) in patients with AS. In the absence of direct evidence from head-to-head randomized controlled trials (RCTs) comparing biologics, individual patient data (IPD) can be matched to determine the relative efficacy of interventions. Propensity score matching (PSM) is a common approach for comparing the effectiveness of two treatments using observational data [20, 21]. Notably, PSMs can leverage open-label IPD to derive indirect comparisons [22], unlike other indirect treatment comparison (ITC) methods based on summary-level data. In this study, PSM techniques were used to evaluate the effectiveness of GOL IV 2 mg/kg compared to IFX 5 mg/kg for the treatment of AS during and beyond initial blinding periods using IPD from the active treatment arms of the GO-ALIVE [18] and ASSERT [14] trials.

## Materials and methods

### Study design and participant characteristics

Details regarding the study design and participant characteristics of the GO-ALIVE [18] and ASSERT [14] trials have been previously reported. Brief descriptions of each trial are provided below. This analysis is based on open-label IPD from each trial for patients randomized to GOL IV 2 mg/kg (*n* = 105) and IFX (*n* = 201).

#### GO-ALIVE

GO-ALIVE (NCT02186873) was a phase 3, randomized, double-blind, placebo-controlled trial that investigated the efficacy and safety of GOL IV 2 mg/kg in active AS. Patients in the placebo group were crossed over to receive GOL IV 2 mg/kg at week 16. The study began in September 2014 and was completed in October 2016, and included 208 adults (≥ 18 years old) who were diagnosed with AS (classified by the modified New York criteria [23]) [18]. Patients had symptoms of active disease (defined as Bath Ankylosing Spondylitis Disease Activity Index [BASDAI] score ≥ 4 and total back pain score ≥ 4), ≥ 0.3 mg/dl concentration of high sensitivity CRP, and intolerance or inadequate response to NSAIDs [18].

#### ASSERT

ASSERT (NCT00207701) was a phase 3, randomized, double-blind, placebo-controlled trial that investigated the efficacy and safety of IFX 5 mg/kg (Remicade®) in active AS. Patients in the placebo group were crossed over to receive IFX 5 mg/kg at week 24. The study began in September 2002 and was completed in February 2005, and included 279 adults (≥ 18 years old) who were diagnosed with AS (defined by the modified New York criteria [23]) [14]. Patients had symptoms of active disease (defined as BASDAI score ≥ 4 and total back pain score ≥ 4), but no CRP threshold was required, as compared to the GO-ALIVE trial.

### Ethics

Janssen is the manufacturer of both products and sponsored the trials that were used in this study. The concept and design for PSM analyses underwent internal Janssen approval and Cornerstone Research Group Inc. was provided access to anonymized data from GO-ALIVE and ASSERT to run the analyses after approval. GO-ALIVE was registered with Clinicaltrials.gov (NCT02186873); ASSERT was conducted before Clinicaltrials.gov was available. The GO-ALIVE protocol was approved by Schulman Associates institutional review board (IRB) for 10 sites in Canada (approval number: 201404734) and the USA (approval number: 201404241); the remaining 36 sites received approval from their local ethics committees. The ASSERT study protocol was reviewed and approved by the respective IRB or independent ethics committee at each site. Each trial was conducted in accordance with the principles of the Declaration of Helsinki and Good Clinical Practices. All patients in both trials were required to give written informed consent before any study-related procedures were performed.

### Propensity score matching

#### Covariates selected for calculating propensity scores

Selection of covariates for propensity score (PS) estimates focus on patient characteristics that may impact the outcome alone or both the outcome and the treatment assigned [20]. Patient characteristics that were available in both clinical trials were identified and ranked a priori (Supplementary Appendix S2). Of the potentially important clinical factors, only smoking could not be used as a PS covariate, because this information was not available in the IPD set for IFX.

#### Propensity score calculation

Propensity scores are defined as the conditional probability of receiving GOL IV, based on covariates that were available in both clinical trials. These were calculated for all patients by fitting a multivariable logistic regression model in which the selected covariates were predictor variables and the treatment received (i.e., GOL IV or IFX) was the dependent variable. All covariates were evaluated at the date of GOL IV treatment initiation in the GO-ALIVE trial and IFX treatment initiation in the ASSERT trial. In the logistic regression model, age, BASDAI, BASFI, Bath Ankylosing Spondylitis Metrology Index (BASMI), CRP, global assessment of disease activity, SF-36 mental and physical component summary scores, inflammation, and total baseline pain were treated as continuous variables, and body mass index (normal weight [BMI < 25]; overweight [25 ≤ BMI < 30]; obese [BMI ≥ 25]), time since diagnosis (< 5 years, ≥ 5 years), gender (male, female), race (caucasian, non-caucasian), global region of residence (North America, Europe, Asia-Pacific, Latin America), and presence of Human Leukocyte Antigen-B27 were treated as dichotomous variables. After fitting the logistic regression model, the logit transform of PS (LTPS) for all patients was stored for subsequent use in establishing matched groups [20].

After estimating PS, the degree of overlap of the patients’ PS from the two treatment groups was assessed. This was conducted by reviewing density plots for LTPS estimates in each treatment group, and by comparing the means, standard deviations (SD), and standardized mean differences (SMDs) of each covariate in each treatment group.

#### Matching of patients using propensity scores

**Fig. 1:**
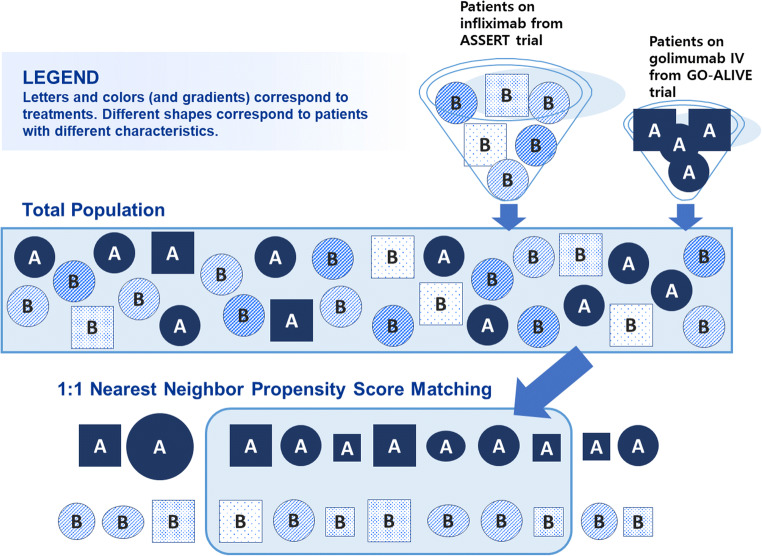
Overview of propensity score matching methods used in this study. *Note*: Active treatment arms were included in the propensity score analysis. The placebo arm was excluded due to crossover

For the primary analysis, patients from the GOL IV group were matched with patients from the IFX group using a 1:1 nearest neighbor (NN) matching algorithm without replacement and with a caliper width of 0.20 SD of the LTPS [24]. Matching without replacement meant that a patient from the GOL IV group who was already matched to a patient from the IFX group was not eligible for matching to another IFX patient. An overview of the PS matching methods used in this study is provided in Fig. 1.

#### Assessment of post-match balance between groups

Differences between treatment groups for each of the selected covariates were compared before and after matching to determine if PSM sufficiently balanced the covariates. Standardized mean differences (SMDs) for the covariates were reviewed to identify imbalances. Thresholds of ≥ 0.10 and ≥ 0.20 for SMDs were used to identify potentially important imbalances, with smaller SMDs indicating a better balance between patient groups [25, 26]. Since estimates derived from PSM are applicable to patients included in the matched sample, the generalizability of estimates from this analysis is assessed by comparing summary statistics between patients included in and excluded from matched sample for the primary outcome (Appendix S1).

### Outcomes and statistical methods

Outcomes of interest were selected a priori in collaboration with external clinical experts: ASAS20, change from baseline in BASFI, and change from baseline in CRP. In the ASSERT and GO-ALIVE studies, ASAS20 response and change from baseline in BASFI scores were included as primary and secondary endpoints, respectively [14, 18]. These are considered to be clinically important, are fully patient reported, and are validated criteria to assess signs and symptoms of AS [27, 28]; therefore, both ASAS20 response and change from baseline in BASFI scores were included as outcomes of interest in the present study.

Change from baseline in CRP levels was included as a secondary endpoint in the ASSERT study [14], though it was not reported in the GO-ALIVE study [18]. In the present analysis, change from baseline in CRP levels was included as an outcome of interest because correlations between CRP levels and both disease activity and functional impairment have previously been reported [29]. In contrast with ASAS20 and BASFI, CRP is an objective parameter of inflammation. In addition, recent network meta-analyses (NMA) in AS have included CRP levels as an outcome of interest [30, 31]. Although change in CRP levels was not originally reported in the GO-ALIVE study, access to IPD allowed for its inclusion in the present analysis.

Outcomes were evaluated from the date of treatment initiation. All statistical analyses were performed using R [32]. Outcomes were reported as odds ratios (OR) for ASAS20 and mean differences (MD) for BASFI and CRP, with 95% confidence intervals (CIs). Odds ratios for ASAS20 were considered statistically significant if the 95% CI did not overlap with 1, and MDs for BASFI and CRP were considered statistically significant if the 95% CI did not overlap with 0.

### Additional analyses

Scenario analyses were conducted to assess the rigor of the primary analysis by incrementally eliminating prognostic variables in order of least importance (Appendix S2). The same algorithm used in the primary analysis (i.e., scenario one) was applied to each subsequent scenario.

Seven sensitivity analyses were conducted to assess the robustness of the primary analysis: (1) 1:1 NN matching without a caliper and without replacement, (2) 1:1 NN matching without a caliper and with replacement, (3) optimal matching, (4) repetition of the primary analysis with an increased caliper width of 0.25 SD of the LTPS, (5) repetition of the primary analysis with missing values imputed by method of last observation carried forward (LOCF), (6) repetition of the primary analysis with missing values imputed by non-responder imputation (NRI), and (7) inverse probability of treatment weighting (IPTW). A structural sensitivity analysis comparing the primary PSM analysis to results obtained using multivariable regression adjustment was also conducted for each outcome (Appendix S5). All sensitivity analyses were implemented using all covariates (scenario one in Appendix S2).

## Results

### Comparison of eligibility criteria between studies

Table 1 reports results of a qualitative comparison of eligibility criteria for the GO-ALIVE and ASSERT trials. Both trials included patients who were diagnosed with AS (defined by the modified New York criteria [23]) [14, 18] and had symptoms of active AS (defined as BASDAI score ≥ 4 and total back pain score ≥ 4), excluded patients who were pregnant, had a serious infection or a sign of malignancy, serious infection, or tuberculosis (based on a chest radiograph taken < 3 months before treatment onset), permitted patients who previously received an anti-TNF (permitted for < 20% of patients recruited to GO-ALIVE and for any patient receiving such therapy > 2 months prior to screening in ASSERT), and permitted concomitant NSAIDs. GO-ALIVE also required patients to have ≥ 0.3 mg/dl concentration of high sensitivity CRP, whereas all patients randomized to infliximab had CRP ≥ 0.3 mg/dl despite ASSERT not having an inclusion criterion based on CRP. Finally, GO-ALIVE required patients to have had an inadequate response, or developed an intolerance to NSAIDs, whereas ASSERT did not. Only 17 (8.5%) patients who were randomized to infliximab discontinued NSAIDs prior to treatment.

**Table 1:** Comparison of eligibility criteria and permitted concomitant therapies in GO-ALIVE and ASSERT

	GO-ALIVE	ASSERT
Inclusion criteria		
Definite ankylosing spondylitis ≥ 3 months based on modified New York radiographic and clinical criteria	Y	Y
Participants have symptoms of active disease as evidenced by both a BASDAI score of ≥ 4 and a VAS score for spinal pain of ≥ 4, each on a scale of 0 to 10 cm.	Y	Y
CRP ≥ 0.3 mg/dl	Y	N^1^
Inadequate response or intolerance to NSAID	Y	N^2^
Complete ankylosis of spine	Y for ≤ 10% of study population	N
Exclusion criteria		
Pregnancy	Y	Y
Participants with chest radiograph within 3 months prior to the first administration of study agent that shows an abnormality suggestive of a malignancy or current active infection, including tuberculosis	Y	Y
Serious infection, hospitalized for infection, or treated with IV antibiotics for infection within 2 months of first administration of study dose	Not permitted (serious infections include but are not limited to hepatitis, pneumonia, sepsis, or pyelonephritis)	Not permitted (serious infections include but are not limited to hepatitis, pneumonia, sepsis, or pyelonephritis)
Prior anti-TNF experience	Allowed in ≤ 20%, except for intravenous golimumab	Allowed > 2 months before screening
Permitted concomitant medications		
Concomitant use of methotrexate (MTX), sulfasalazine (SSZ), hydroxychloroquine, systemic corticosteroids	Low dose oral corticosteroids permitted	MTX and SSZ not permitted within 2 weeks of screening; DMARDs other than MTX and SSZ not permitted within 6 months of screening; systemic corticosteroids not permitted within 4 months of screening.
NSAIDs	Y	Y
Acetaminophen	NR	Y
Tramadol	NR	Y
Cytotoxic drugs	NR	N

*BASDAI* Bath Ankylosing Spondylitis Disease Activity Index, *CRP* C-reactive protein, *DMARD* disease-modifying anti-rheumatic drug, *IV* intravenous, *N* no, *NR* not reported, *NSAID* non-steroidal anti-inflammatory drug, *TNF* tumor necrosis factor, *VAS* visual analog scale, *Y* yes

^1^All patients randomized to infliximab had CRP ≥ 0.3 mg/dl at screening.

^2^Only 17 (8.5%) of patients randomized to infliximab discontinued NSAIDs before treatment.

### Intravenous golimumab and infliximab patients eligible for PSM and post-match balance between groups.

In total, 105 AS patients were treated with GOL IV in the GO-ALIVE trial [18] and 201 patients were treated with IFX in the ASSERT trial [14]. After matching on all possible covariates, a total of 118 patients were included in the ASAS20 analysis (*n* = 59 in each treatment group), 96 patients in the BASFI analysis (*n* = 48 in each treatment group), and 160 patients in the CRP analysis (*n* = 80 in each treatment group; see scenario one in Appendix S2). Table 2 presents patient characteristics of all 16 covariates before and after matching for the ASAS20 analysis. Before matching, 86% of covariates had imbalances across treatment groups, with SMDs ≥ 0.10. Patients who received GOL IV had on average lower baseline BASMI scores (SMD = 0.67), higher SF-36 MCS scores (SMD = 0.57), and longer time since diagnosis (SMD = 0.54) than patients who received IFX. Matching yielded large improvements in covariate balance across treatment groups, with 74% of covariates having imbalances at the ≤ 0.10 SMD threshold and all covariates with imbalances at the ≤ 0.20 threshold.

**Table 2:** Baseline characteristics by treatment group before and after propensity score matching on all 16 ASAS20 covariates

	Pre-match			Post-match		
Baseline characteristic	Infliximab	Golimumab IV	SMD	Infliximab	Golimumab IV	SMD
Age in years, mean (SD)	39.6 (10.6)	38.4 (10.1)	*0.113*	39 (10.2)	38.4 (10.1)	0.057
Male, *N* (%)	157 (78.1%)	86 (81.9%)	0.095	49 (83.1%)	48 (81.4%)	0.044
HLA-B27, *N* (%)	173 (86.1%)	94 (89.5%)	*0.105*	53 (89.8%)	52 (88.1%)	0.054
Methotrexate use, *N* (%)	18 (9%)	16 (15.2%)	*0.193*	6 (10.2%)	7 (11.9%)	0.054
CRP, mean (SD)	2.4 (2.7)	2 (1.8)	*0.178*	1.8 (1.3)	2 (2)	*0.106*
BASMI, mean (SD)	4 (2)	5 (0.9)	*0.671*	5.1 (1.7)	4.9 (0.9)	*0.157*
SF-36 physical component summary score, mean (SD)	29 (7.3)	32.4 (5.6)	*0.523*	31.3 (7)	31.6 (5)	0.049
Inflammation, mean (SD)	6.9 (2.3)	7.3 (1.5)	*0.219*	7 (2.3)	7.1 (1.4)	0.021
Caucasian, *N* (%)	197 (98%)	89 (84.8%)	*0.484*	59 (100%)	58 (98.3%)	*0.184*
BASFI, mean (SD)	5.7 (1.9)	6.3 (1.9)	*0.292*	6.3 (1.5)	6.2 (1.6)	*0.105*
Disease duration ≥ 5 years, *N* (%)	129 (64.2%)	40 (38.1%)	*0.539*	30 (50.8%)	27 (45.8%)	*0.101*
Global assessment of disease activity, mean (SD)	6.8 (1.8)	7.3 (1.3)	*0.316*	7.1 (1.8)	7.3 (1.2)	0.084
BASDAI, mean (SD)	6.5 (1.5)	7.1 (1.2)	*0.406*	6.7 (1.5)	6.8 (1.1)	0.091
SF-36 mental component summary score, mean (SD)	46.1 (10.9)	40 (10.4)	*0.571*	41.2 (11.3)	41.1 (9.6)	0.014
Body mass index, *N* (%)						
Normal weight	85 (42.3%)	44 (41.9%)	0.008	21 (35.6%)	23 (39%)	0.070
Overweight	93 (46.3%)	37 (35.2%)	*0.225*	25 (42.4%)	24 (40.7%)	0.034
Obese	23 (11.4%)	24 (22.9%)	*0.305*	13 (22%)	12 (20.3%)	0.041
Region, *N* (%)						
North America	43 (21.4%)	9 (8.6%)	*0.364*	7 (11.9%)	7 (11.9%)	0.000
Europe	158 (78.6%)	83 (79%)	0.011	52 (88.1%)	52 (88.1%)	0.000
Asia/Pacific	0 (0%)	10 (9.5%)	*0.457*	0 (0%)	0 (0%)	NA
Latin America	0 (0%)	3 (2.9%)	*0.241*	0 (0%)	0 (0%)	NA
Balance diagnostics across all baseline characteristics	Mean of SMDs: 0.301			Mean of SMDs: 0.067		
	Proportion of SMDs ≥ 0.10: 86%			Proportion of SMDs ≥ 0.10: 26%		
	Proportion of SMDs ≥ 0.20: 67%			Proportion of SMDs ≥ 0.20: 0%		

Note: Summary statistics are reported before and after 1:1 nearest neighbor matching without replacement and caliper of 0.2 standard deviations of the logit transform of the propensity score. The matching algorithm includes all covariates considered prognostic of ASAS20, regardless of their clinical ranking. Italic values denote SMDs ≥ 0.10. Covariates used in the matching algorithm for change from baseline in BASFI included total baseline pain and all ASAS20 covariates except for body mass index. Covariates used in the matching algorithm for change from baseline in CRP included all ASAS20 covariates except for methotrexate use, BASMI, SF-36 physical and mental component summary scores, region, and race. Please refer to Supplementary Appendix S2 for a complete listing of covariates for each outcome. Inflammation refers to the average of questions 5 (morning stiffness duration) and 6 (morning stiffness severity) of the BASDAI.

*BASDAI* Bath Ankylosing Spondylitis Disease Activity Index, *BASFI* Bath Ankylosing Spondylitis Functional Index, *BASMI* Bath Ankylosing Spondylitis Metrology Index, *CRP* C-reactive protein, *HLA* human leukocyte antigen, *SMD* standardized mean difference

**Fig. 2:**
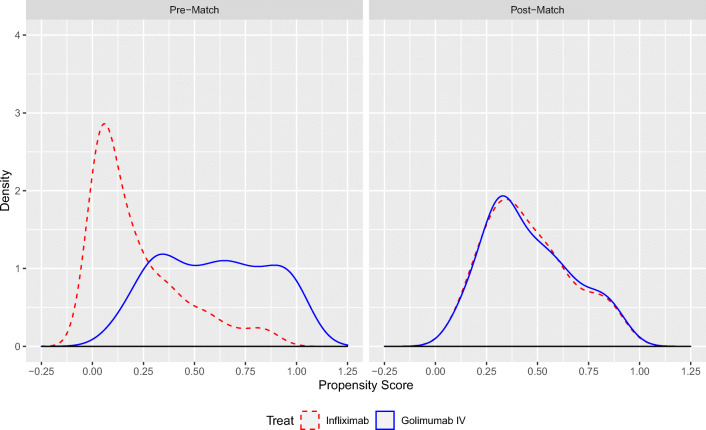
Propensity score balance before and after matching on all 16 ASAS20 covariates. Abbreviations: ASAS20 = improvement of ≥ 20% in the Assessment of Spondyloarthritis International Society Criteria. *Note*: The figure shows propensity score density plots before and after matching on all 16 covariates included in the ASAS20 matching algorithm. Please refer to Table 2 for a list of covariates. The matching algorithm was 1:1 nearest neighbor matching without replacement and a caliper of 0.2 standard deviations of the logit transform of the propensity score.

Density plots of the PS estimates before and after 1:1 NN matching are presented in Fig. 2 for ASAS20 response, and in Appendix S3 for change from baseline in BASFI score and for change from baseline in CRP levels. Before matching, the estimated probabilities of receiving GOL IV, as predicted from baseline patient characteristics, were systematically larger among patients who received GOL IV than those who received IFX for all outcomes. After matching on the LTPS, the distribution of predicted probabilities of receiving GOL IV was nearly identical across treatment groups for all outcomes.

A comparison of baseline summary statistics for patients included in and excluded from the matched sample is presented in Appendix S1, based on the primary 1:1 NN matching algorithm for ASAS20. In IFX-treated patients, the matched sample had on average higher BASMI (SMD = 0.91), higher BASFI (SMD = 0.61), and lower SF-36 MCS (SMD = 0.77) than unmatched patients. For GOL IV-treated patients, the matched sample had on average lower BASMI (SMD = 0.43), longer disease duration (SMD = 0.53), higher SF-36 MCS (SMD = 0.40), and more Caucasians (SMD = 0.77) than unmatched patients.

### ASAS20 response

**Fig. 3:**
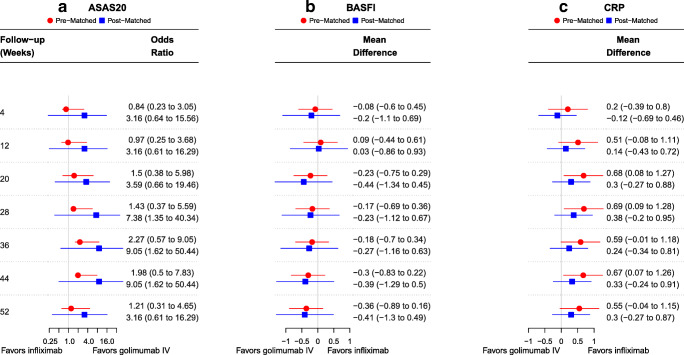
Relative efficacy of intravenous golimumab compared to infliximab over 52 weeks before and after propensity score matching. Achievement of ASAS20 response and decrements from baseline in BASFI or CRP are considered favorable outcomes. For ASAS20, odds ratios > 1 (< 1) denote greater (lesser) efficacy of golimumab compared to infliximab. For BASFI and CRP, mean differences < 0 (> 0) denote greater (lesser) efficacy of golimumab compared to infliximab. Abbreviations: ASAS20 = improvement of ≥ 20% in the Assessment of Spondyloarthritis International Society Criteria; BASFI = Bath Ankylosing Spondylitis Functional Index; CRP = C-reactive protein. *Note:* The figure above displays forest plots of the relative efficacy of golimumab compared to infliximab for **a** ASAS20, **b** change in BASFI, and **c** change in CRP before and after 1:1 nearest neighbor matching without replacement and a caliper of 0.2 standard deviations of the logit transform of the propensity score. The matching algorithm includes all ranked covariates for each outcome. Please refer to Supplementary Appendix S2 for a complete listing of covariates for each outcome

Before matching, GOL IV showed no difference in ASAS20 response compared to IFX for all time points assessed. After matching, GOL IV showed significantly greater odds of ASAS20 response than IFX for weeks 28, 36, and 44 (OR = 9.05 [95% CI 1.62 to 50.4] at week 44; Fig. 3a). No differences were observed between treatments at the remaining time points.

### Change from baseline in BASFI

Before matching, changes from baseline in BASFI scores were similar for GOL IV and IFX at all time points; however, there was a trend towards greater change from baseline in BASFI scores over time with GOL IV than with IFX. After matching, this trend remained over the 52-week follow-up (MD = − 0.41 [95% CI − 1.3 to 0.49] at week 52; Fig. 3b), although the point estimate did not reach the minimum clinically important improvement (MCII) cutoff of 0.6 for BASFI in patients with active AS [33].

### Change from baseline in CRP

Before matching, change from baseline in CRP levels was significantly greater for IFX than GOL IV at weeks 20, 36, and 44 (MD = 0.67 [95% CI 0.07 to 1.26] at week 44). No differences were observed between the two treatments at the remaining timepoints (MD = 0.55 [95% CI − 0.04 to 1.15] at week 52). After matching, the change from baseline in CRP levels was not statistically different between GOL IV and IFX at all timepoints (MD = 0.30 [95% CI − 0.27 to 0.87] at week 52; Fig. 3c).

### Additional analyses

Scenario analyses reducing number of characteristics adjusted for produced results that were similar to the primary analysis (Appendix S2). Similarly, sensitivity analyses using various matching and weighting algorithms produced results that were similar to primary analysis (Appendix S4). Multivariable regression models generally yielded somewhat smaller and more precise effect estimates in favor of GOL IV versus IFX (Appendix S5).

For both ASAS20 and BASFI, matching or adjusting for BASMI produced a numeric change in effect estimates in favor of GOL IV. BASMI was on average higher in GOL IV-treated patients than in IFX-related patients, possibly because GO-ALIVE was initiated > 10 years after ASSERT in patients who may have had more active or advanced disease. Notably, BASMI was associated with lower ASAS20 response in the pooled sample used for this analysis (unadjusted OR [95% CI] = 0.36 [0.23 to 0.57]; not reported), suggesting that PSM was effective in reducing bias that initially favored IFX-treated patients through lower BASMI levels compared with patients who were treated with GOL IV.

For ASAS20 response, sensitivity analyses showed that GOL IV was significantly more efficacious than IFX at weeks 28–44 (Appendix S4), with some showing significant improvement as early as week 20 and as late as week 52. The point estimates were larger and the 95% CIs were more narrow when using IPTW-derived treatment effects as well as NN matching with imputed outcomes, compared with the primary analyses. For example, the IPTW-derived estimate of ASAS20 response was significantly greater for GOL IV than IFX at weeks 28 (OR = 5.74 [95% CI 1.62 to 20.37]), 36 (OR = 11.24 [95% CI 3.05 to 41.47]), 44 (OR = 9.54 [95% CI 2.6 to 34.93]), and 52 (OR = 5.8 [95% CI 1.63 to 20.68]). Similarly, for both BASFI and CRP, the comparative efficacy of GOL IV vs IFX was more favorable when using IPTW or NN matching with imputation (Appendices S3).

## Discussion

In the absence of randomized head-to-head comparative trial data, PSM is a valuable method that enables a pairwise comparison of two treatments from separate studies using IPD [20, 34]. This study used PSM methods to generate matched comparisons of GOL IV and IFX to assess their relative efficacy in terms of ASAS20 response and change from baseline in BASFI scores and CRP levels in patients with AS. After matching, treatment with GOL IV was associated with significantly greater odds of improvement in ASAS20 response than IFX. These effects were particularly robust at weeks 28, 36, and 44. Treatment with GOL IV was also associated with greater numerical improvements from baseline in BASFI score than IFX, though these changes did not reach statistical significance or the MCII cutoff of 0.6 for BASFI in patients with active AS [33].

In contrast, IFX treatment was associated with greater numerical reductions from baseline in CRP levels than GOL IV, though also without statistical significance. Results of the scenario analyses were consistent with the findings of the primary analyses. Findings were also robust across sensitivity analyses for all three outcomes. Notably, the comparative efficacy of GOL IV versus IFX was more pronounced using NN matching with imputation of missing outcomes and with IPTW estimation.

Several recent ITC studies have compared the effectiveness of biologic therapies in AS at time points varying from 6 to 24 weeks of treatment, and have generally concluded that no specific agent is superior to others [8, 30, 31, 35–38]. At the substantially longer treatment time points included in the present PSM study, GOL IV was found to be significantly more efficacious than IFX in terms of ASAS20 response. Collectively, these findings could suggest that GOL IV has sustained benefits in efficacy compared with IFX that become apparent upon continued treatment at weeks 28–44 of follow-up.

Among the most notable strengths of the present study is that it is the first of its kind to compare any two biologics in the treatment of active AS using PSM. We were able to compare GOL IV and IFX over a long-term follow-up period leveraging IPD from the ASSERT [14] and GO-ALIVE [18] trials. Access to both IPD sets allowed matching of patients between trials, and rigorous assessment of the comparative efficacy of GOL IV and IFX in the absence of a head-to-head trial. Although ITCs may be derived from aggregate data using network meta-analyses, such estimates may be biased by trial-level heterogeneity. In contrast, IPD provides more flexibility in adjusting for heterogeneity using standard covariate-adjustment, matching, or reweighting techniques.

When only one IPD set is available and summary-level data are available for the comparator trial, a matching-adjusted indirect comparison (MAIC) can be performed. Though MAIC is an acceptable approach, the absence of IPD from one of the trials necessitates the use of a broader IPD population compared with the comparator study. Weighting techniques are required to align patient populations, which may result in a smaller effective sample size [30]. In contrast, the PSM methods used in the present study use matching methods that are more intuitive to clinicians and decision makers compared with weighting methods. Propensity score matching methods also have more flexibility to adjust for differences in patient characteristics between trials and are therefore preferable when IPD can be accessed for both studies. In addition to the novelty and significance of the results themselves, the robustness of the findings is supported by the results of the numerous sensitivity and scenario analyses. Seven alternative algorithms in the sensitivity analysis for ASAS20 response produced very similar findings to those of the primary analysis, and scenario analyses with 7–16 covariates that were available in both clinical trials resulted in significant favorability for GOL IV over IFX at longer treatment times.

One limitation of this study is the difficulty of incorporating all factors that may influence treatment effects and prognoses. To account for this, covariates that were available in both studies were identified and ranked based on prognostic importance a priori, which enabled adjustment of 16 factors between the treatment groups. However, as with any analysis that incorporates multivariable modeling or matching techniques, it is impossible to rule out unmeasured confounders and/or those that were not captured in both datasets that may influence patient outcomes (e.g., symptom duration, smoking, joint damage at baseline, and inadequate response or intolerance to NSAIDs in this study). In addition, there may be limitations associated with time since diagnosis as a covariate, since duration of symptoms is not captured by this measure. Though information on symptom duration was not available in the IPD set, it is reassuring that the scenario analysis removing disease duration did not alter results. The use of NN caliper matching to IPD from the active arms of GO-ALIVE and ASSERT resulted in relatively small analytic samples that may be underpowered compared to the original RCTs in their ability to detect small but relevant clinical effects. It is therefore unsurprising that the smaller effect sizes estimated at weeks 4, 12, 20, and 52 were not statistically significant. Although this must be considered when interpreting the results of this study, the primary analyses incorporated a substantial list of covariates, and pre-matching imbalances were effectively addressed by PSM. Furthermore, the results of the primary analysis were consistent with effect estimates derived from several other PS algorithms (such as IPTW) and multivariable regression using larger sample sizes. Finally, different assays may have been used to measure CRP levels in the original studies. Although CRP measurements were standardized for units in this study, the use of different assays warrants caution when interpreting the results of change from baseline in CRP levels.

## Conclusions

This is the first analysis of its kind to leverage clinical trial data to compare two biologics using PS methods in the treatment of active AS. Overall, these results suggest that GOL IV 2 mg/kg may be associated with a significantly greater ASAS20 response than IFX 5 mg/kg in patients with AS after 28–44 weeks of treatment. In the absence of head-to-head studies of biologics in the treatment of AS, comparative effectiveness can be assessed by PSM.

## Electronic supplementary material

ESM1 (DOCX 222 kb)

ESM2 (PDF 32.8 kb)

## References

[1] Gorman JD, Sack KE, Davis JC (2002). Treatment of ankylosing spondylitis by inhibition of tumor necrosis factor alpha. N Engl J Med.

[2] Garcia-Montoya L, Gul H, Emery P (2018) Recent advances in ankylosing spondylitis: understanding the disease and management. F1000Res 7. 10.12688/f1000research.14956.1 10.12688/f1000research.14956.1 PMC6173104 30345001

[3] Ward MM, Deodhar A, Akl EA, Lui A, Ermann J, Gensler LS, Smith JA, Borenstein D, Hiratzka J, Weiss PF, Inman RD, Majithia V, Haroon N, Maksymowych WP, Joyce J, Clark BM, Colbert RA, Figgie MP, Hallegua DS, Prete PE, Rosenbaum JT, Stebulis JA, van den Bosch F, Yu DT, Miller AS, Reveille JD, Caplan L (2016). American College of Rheumatology/Spondylitis Association of America/Spondyloarthritis Research and Treatment Network 2015 recommendations for the treatment of ankylosing spondylitis and nonradiographic axial spondyloarthritis. Arthritis Rheumatol.

[4] Zink A, Braun J, Listing J, Wollenhaupt J (2000). Disability and handicap in rheumatoid arthritis and ankylosing spondylitis--results from the German rheumatological database. German Collaborative Arthritis Centers. J Rheumatol.

[5] Gran JT, Skomsvoll JF (1997). The outcome of ankylosing spondylitis: a study of 100 patients. Br J Rheumatol.

[6] Ward MM (1999). Health-related quality of life in ankylosing spondylitis: a survey of 175 patients. Arthritis Care Res.

[7] Taylor AL, Balakrishnan C, Calin A (1998). Reference centile charts for measures of disease activity, functional impairment, and metrology in ankylosing spondylitis. Arthritis Rheum.

[8] Chen C, Zhang X, Xiao L, Zhang X, Ma X (2016). Comparative effectiveness of biologic therapy regimens for ankylosing spondylitis: a systematic review and a network meta-analysis. Medicine (Baltimore).

[9] Bao C, Huang F, Khan MA, Fei K, Wu Z, Han C, Hsia EC (2014). Safety and efficacy of golimumab in Chinese patients with active ankylosing spondylitis: 1-year results of a multicentre, randomized, double-blind, placebo-controlled phase III trial. Rheumatology (Oxford).

[10] van der Heijde D, Ramiro S, Landewé R, Baraliakos X, Van den Bosch F, Sepriano A, Regel A, Ciurea A, Dagfinrud H, Dougados M, van Gaalen F, Géher P, van der Horst-Bruinsma I, Inman RD, Jongkees M, Kiltz U, Kvien TK, Machado PM, Marzo-Ortega H, Molto A, Navarro-Compàn V, Ozgocmen S, Pimentel-Santos FM, Reveille J, Rudwaleit M, Sieper J, Sampaio-Barros P, Wiek D, Braun J (2017). 2016 update of the ASAS-EULAR management recommendations for axial spondyloarthritis. Ann Rheum Dis.

[11] Baeten D, Baraliakos X, Braun J, Sieper J, Emery P, van der Heijde D, McInnes I, van Laar JM, Landewe R, Wordsworth P, Wollenhaupt J, Kellner H, Paramarta J, Wei J, Brachat A, Bek S, Laurent D, Li Y, Wang YA, Bertolino AP, Gsteiger S, Wright AM, Hueber W (2013). Anti-interleukin-17A monoclonal antibody secukinumab in treatment of ankylosing spondylitis: a randomised, double-blind, placebo-controlled trial. Lancet (London, England).

[12] McInnes IB, Mease PJ, Kirkham B, Kavanaugh A, Ritchlin CT, Rahman P, van der Heijde D, Landewe R, Conaghan PG, Gottlieb AB, Richards H, Pricop L, Ligozio G, Patekar M, Mpofu S (2015). Secukinumab, a human anti-interleukin-17A monoclonal antibody, in patients with psoriatic arthritis (FUTURE 2): a randomised, double-blind, placebo-controlled, phase 3 trial. Lancet (London, England).

[13] Deodhar A, Poddubnyy D, Pacheco-Tena C, Salvarani C, Lespessailles E, Rahman P, Jarvinen P, Sanchez-Burson J, Gaffney K, Lee EB, Krishnan E, Santisteban S, Li X, Zhao F, Carlier H, Reveille JD (2019). Efficacy and safety of ixekizumab in the treatment of radiographic axial spondyloarthritis: sixteen-week results from a phase iii randomized, double-blind, placebo-controlled trial in patients with prior inadequate response to or intolerance of tumor necrosis factor inhibitors. Arthritis Rheumatol.

[14] van der Heijde D, Dijkmans B, Geusens P, Sieper J, DeWoody K, Williamson P, Braun J, Ankylosing Spondylitis Study for the Evaluation of Recombinant Infliximab Therapy Study G (2005). Efficacy and safety of infliximab in patients with ankylosing spondylitis: results of a randomized, placebo-controlled trial (ASSERT). Arthritis Rheum.

[15] Braun J, Brandt J, Listing J, Zink A, Alten R, Golder W, Gromnica-Ihle E, Kellner H, Krause A, Schneider M, Sorensen H, Zeidler H, Thriene W, Sieper J (2002). Treatment of active ankylosing spondylitis with infliximab: a randomised controlled multicentre trial. Lancet (London, England).

[16] Brandt J, Haibel H, Cornely D, Golder W, Gonzalez J, Reddig J, Thriene W, Sieper J, Braun J (2000). Successful treatment of active ankylosing spondylitis with the anti-tumor necrosis factor alpha monoclonal antibody infliximab. Arthritis Rheum.

[17] Grainger R, Harrison AA (2007). Infliximab in the treatment of ankylosing spondylitis. Biologics.

[18] Deodhar A, Reveille JD, Harrison DD, Kim L, Lo KH, Leu JH, Hsia EC (2018). Safety and efficacy of golimumab administered intravenously in adults with ankylosing spondylitis: results through week 28 of the GO-ALIVE study. J Rheumatol.

[19] Janssen Biotech Inc (2018) Medication Guide: Simponi Aria. Last updated: May, 2018. Last accessed: January 17, 2019

[20] Austin PC (2011). An introduction to propensity score methods for reducing the effects of confounding in observational studies. Multivar Behav Res.

[21] Stuart EA (2010). Matching methods for causal inference: a review and a look forward. Stat Sci.

[22] Mann H, Andersohn F, Bodnar C, Mitsudomi T, Mok TSK, Yang JC, Hoyle C (2018). Adjusted indirect comparison using propensity score matching of osimertinib to platinum-based doublet chemotherapy in patients with EGFRm T790M NSCLC who have progressed after EGFR-TKI. Clin Drug Investig.

[23] van der Linden S, Valkenburg HA, Cats A (1984). Evaluation of diagnostic criteria for ankylosing spondylitis. A proposal for modification of the New York criteria. Arthritis Rheum.

[24] Austin PC (2011). Optimal caliper widths for propensity-score matching when estimating differences in means and differences in proportions in observational studies. Pharm Stat.

[25] Austin PC (2009). Balance diagnostics for comparing the distribution of baseline covariates between treatment groups in propensity-score matched samples. Stat Med.

[26] Stuart EA, Lee BK, Leacy FP (2013). Prognostic score-based balance measures can be a useful diagnostic for propensity score methods in comparative effectiveness research. J Clin Epidemiol.

[27] van der Heijde D, Dougados M, Davis J, Weisman MH, Maksymowych W, Braun J, Hallegua DS, Bruckel J (2005). ASsessment in Ankylosing Spondylitis International Working Group/Spondylitis Association of America recommendations for conducting clinical trials in ankylosing spondylitis. Arthritis Rheum.

[28] Landewe R, van Tubergen A (2015). Clinical tools to assess and monitor spondyloarthritis. Curr Rheumatol Rep.

[29] Benhamou M, Gossec L, Dougados M (2009). Clinical relevance of C-reactive protein in ankylosing spondylitis and evaluation of the NSAIDs/coxibs’ treatment effect on C-reactive protein. Rheumatology.

[30] Deodhar A, Chakravarty SD, Cameron C, Peterson S, Hensman R, Fogarty S, Spin P, Kafka S, Nair S, Gensler LS (2020). A systematic review and network meta-analysis of current and investigational treatments for active ankylosing spondylitis. Clin Rheumatol. 10.1007/s10067-020-04970-3 10.1007/s10067-020-04970-3 PMC7338808 32107666

[31] Wang R, Dasgupta A, Ward MM (2018). Comparative efficacy of tumor necrosis factor-alpha inhibitors in ankylosing spondylitis: a systematic review and Bayesian network metaanalysis. J Rheumatol.

[32] R Core Team (2013) R: A language and environment for statistical computing. R foundation for statistical computing, Vienna, Austria. Available at: Http://www.R-project.Org/. Accessed 1 Dec 2018

[33] Kviatkovsky MJ, Ramiro S, Landewe R, Dougados M, Tubach F, Bellamy N, Hochberg M, Ravaud P, Martin-Mola E, Awada H, Bombardier C, Felson D, Hajjaj-Hassouni N, Logeart I, Matucci-Cerinic M, van de Laar M, van der Heijde D (2016). The minimum clinically important improvement and patient-acceptable symptom state in the BASDAI and BASFI for patients with ankylosing spondylitis. J Rheumatol.

[34] Little RJ, Rubin DB (2000). Causal effects in clinical and epidemiological studies via potential outcomes: concepts and analytical approaches. Annu Rev Public Health.

[35] Migliore A, Bizzi E, Bernardi M, Picchianti Diamanti A, Lagana B, Petrella L (2015). Indirect comparison between subcutaneous biologic agents in ankylosing spondylitis. Clinical drug investigation.

[36] Shu T, Chen GH, Rong L, Feng F, Yang B, Chen R, Wang J (2013). Indirect comparison of anti-TNF-alpha agents for active ankylosing spondylitis: mixed treatment comparison of randomized controlled trials. Clin Exp Rheumatol.

[37] Baji P, Pentek M, Szanto S, Geher P, Gulacsi L, Balogh O, Brodszky V (2014). Comparative efficacy and safety of biosimilar infliximab and other biological treatments in ankylosing spondylitis: systematic literature review and meta-analysis. Eur J Health Econ.

[38] Liu W, Wu YH, Zhang L, Liu XY, Bin X, Bin L, Wang Y, Ji Y (2016). Efficacy and safety of TNF-alpha inhibitors for active ankylosing spondylitis patients: multiple treatment comparisons in a network meta-analysis. Sci Rep.

[39] Signorovitch JE, Sikirica V, Erder MH, Xie J, Lu M, Hodgkins PS, Betts KA, Wu EQ (2012). Matching-adjusted indirect comparisons: a new tool for timely comparative effectiveness research. Value Health.

