# Supplementary material for: Propensity score matching/reweighting analysis comparing intravenous golimumab to infliximab for ankylosing spondylitis using data from the GO-ALIVE and ASSERT trials
Source: Clin Rheumatol. 2020 May 4;39(10):2907–17. doi: 10.1007/s10067-020-05051-1 (PMC7497341; doi:10.1007/s10067-020-05051-1)
Supplement: Supplementary file 1 — (DOCX 222 kb) [file 10067_2020_5051_MOESM1_ESM.docx]

**Appendix S1**

Baseline summary statistics by inclusion in the propensity score matched sample for ASAS20.

|  | **Infliximab** | | | **Golimumab IV** | | |
| --- | --- | --- | --- | --- | --- | --- |
|  | Matched | Unmatched | SMD | Matched | Unmatched | SMD |
| Age in years, mean (SD) | 39.0+/-10.2 | 39.3+/-11.1 | 0.04 | 38.4+/-10.1 | 38.6+/-9.5 | 0.02 |
| Male, N (%) | 49 (83%) | 87 (76%) | **0.18** | 48 (81%) | 28 (85%) | 0.09 |
| Human leukocyte antigen B27, N (%) | 53 (90%) | 96 (83%) | **0.19** | 52 (88%) | 31 (94%) | **0.20** |
| Methotrexate use, N (%) | 6 (10%) | 7 (6%) | **0.15** | 7 (12%) | 6 (18%) | **0.18** |
| CRP, mean (SD) | 1.8+/-1.3 | 2.8+/-3.0 | **0.43** | 2.0+/-2.0 | 2.0+/-1.5 | 0.03 |
| BASMI, mean (SD) | 5.1+/-1.7 | 3.4+/-1.9 | **0.91** | 4.9+/-0.9 | 5.2+/-0.9 | **0.43** |
| SF-36 physical component score, mean (SD) | 31.3+/-7.0 | 28.3+/-7.1 | **0.43** | 31.6+/-5.0 | 34.5+/-6.1 | **0.51** |
| Inflammation, mean (SD) | 7.0+/-2.3 | 6.7+/-2.2 | **0.15** | 7.1+/-1.4 | 7.5+/-1.4 | **0.33** |
| Caucasian, N (%) | 59 (100%) | 111 (97%) | **0.27** | 58 (98%) | 24 (73%) | **0.77** |
| BASFI, mean (SD) | 6.3+/-1.5 | 5.3+/-1.8 | **0.61** | 6.2+/-1.6 | 6.4+/-2.4 | **0.10** |
| Disease duration ≥5 Years, N (%) | 30 (100%) | 82 (97%) | **0.43** | 27 (98%) | 7 (73%) | **0.53** |
| Global assessment of disease activity, mean (SD) | 7.1+/-1.8 | 6.6+/-1.8 | **0.29** | 7.3+/-1.2 | 7.4+/-1.5 | **0.13** |
| BASDAI, mean (SD) | 6.7+/-1.5 | 6.3+/-1.5 | **0.27** | 6.8+/-1.1 | 7.3+/-1.2 | **0.40** |
| SF-36 mental component score, mean (SD) | 41.2+/-11.3 | 49.2+/-9.4 | **0.77** | 41.1+/-9.6 | 37.0+/-11.1 | **0.40** |
| **Body Mass Index, N (%)** |  |  |  |  |  |  |
| Normal Weight | 21 (36%) | 54 (47%) | **0.23** | 23 (39%) | 15 (45%) | **0.13** |
| Overweight | 25 (42%) | 57 (50%) | **0.14** | 24 (41%) | 11 (33%) | **0.15** |
| Obese | 13 (22%) | 4 (3%) | **0.57** | 12 (20%) | 7 (21%) | 0.02 |
| **Region, N (%)** |  |  |  |  |  |  |
| North America | 7 (12%) | 26 (23%) | **0.29** | 7 (12%) | 0 (0%) | **0.51** |
| Europe | 52 (88%) | 89 (77%) | **0.29** | 52 (88%) | 25 (76%) | **0.32** |
| Asia/Pacific | 0 (0%) | 0 (0%) | NE | 0 (0%) | 1 (3%) | **0.25** |
| Latin America | 0 (0%) | 0 (0%) | NE | 0 (0%) | 7 (21%) | **0.72** |
| Sample size | 59 | 115 | - | 59 | 32 | - |
| Mean of SMDs | 0.349 | | | 0.296 | | |
|  |  | | |  | | |
| Proportion of SMDs ≥0.10 (%) | 94.74 | | | 80.95 | | |
| Proportion of SMDs ≥0.20 (%) | 68.42 | | | 61.90 | | |

Note: Summary statistics reported matched and unmatched patients based on 1:1 nearest neighbor matching without replacement and caliper of 0.2 standard deviations of the logit transform of the propensity score. The matching algorithm includes all covariates considered prognostic of ASAS20, regardless of their clinical ranking. Bold font denotes SMDs ≥0.10. Covariates used in the matching algorithm for change from BASFI included total baseline pain and all ASAS20 covariates except for Body Mass Index. Covariates used in the matching algorithm for change in CRP included all ASAS20 covariates except for methotrexate use, BASMI, SF-36 physical component score, SF-36 mental component score, region, and race.

Abbreviations: BASDAI = Bath Ankylosing Spondylitis Disease Activity Index; BASFI = Bath Ankylosing Spondylitis Functional Index; BASMI = Bath Ankylosing Spondylitis Metrology Index; CRP = C-Reactive Protein; HLA = Human Leukocyte Antigen; SMD = Standardized Mean Difference.

**Appendix S2**

ASAS20: *A priori* rankings of prognostic factors and treatment effect modifiers. The table below includes post-matching balance diagnostics under each scenario after implementing 1:1 nearest neighbor matching without replacement and a caliper of 0.2 standard deviations of the logit transform of the propensity score.

|  | **Scenarios** | | | | | | | | | | | | | | | | |
| --- | --- | --- | --- | --- | --- | --- | --- | --- | --- | --- | --- | --- | --- | --- | --- | --- | --- |
| **Factors ordered by *a priori* rankings** | **1** | **2** | **3** | **4** | **5** | **6** | **7** | **8** | **9** | **10** | **11** | **12** | **13** | **14** | **15** | **16** |  |
| Age | X | X | X | X | X | X | X | X | X | X | X | X | X | X | X | X |  |
| Gender | X | X | X | X | X | X | X | X | X | X | X | X | X | X | X |  |  |
| Body mass index | X | X | X | X | X | X | X | X | X | X | X | X | X | X |  |  |  |
| Human leukocyte antigen-B27 | X | X | X | X | X | X | X | X | X | X | X | X | X |  |  |  |  |
| Methotrexate use | X | X | X | X | X | X | X | X | X | X | X | X |  |  |  |  |  |
| CRP | X | X | X | X | X | X | X | X | X | X | X |  |  |  |  |  |  |
| BASMI | X | X | X | X | X | X | X | X | X | X |  |  |  |  |  |  |  |
| SF-36 physical component score | X | X | X | X | X | X | X | X | X |  |  |  |  |  |  |  |  |
| Region | X | X | X | X | X | X | X | X |  |  |  |  |  |  |  |  |  |
| Inflammation | X | X | X | X | X | X | X |  |  |  |  |  |  |  |  |  |  |
| Race | X | X | X | X | X | X |  |  |  |  |  |  |  |  |  |  |  |
| BASFI | X | X | X | X | X |  |  |  |  |  |  |  |  |  |  |  |  |
| Disease duration | X | X | X | X |  |  |  |  |  |  |  |  |  |  |  |  |  |
| Global assessment of disease activity | X | X | X |  |  |  |  |  |  |  |  |  |  |  |  |  |  |
| BASDAI | X | X |  |  |  |  |  |  |  |  |  |  |  |  |  |  |  |
| SF-36 mental component score | X |  |  |  |  |  |  |  |  |  |  |  |  |  |  |  |  |
| Sample Size | 118 | 118 | 120 | 134 | 136 | 146 | 144 | 138 | 140 | 154 | 178 | 180 | 186 | 184 | 192 | 192 |  |
| Mean of SMDs | 0.07 | 0.07 | 0.09 | 0.12 | 0.15 | 0.17 | 0.2 | 0.22 | 0.27 | 0.25 | 0.25 | 0.25 | 0.26 | 0.3 | 0.28 | 0.3 |  |
| Proportion of SMDs ≥0.10 (%) | 26.32 | 15.79 | 42.11 | 42.11 | 42.11 | 47.37 | 52.63 | 42.11 | 66.67 | 66.67 | 71.43 | 61.9 | 71.43 | 66.67 | 85.71 | 76.19 |  |
| Proportion of SMDs ≥0.20 (%) | 0.00 | 0.00 | 0 | 15.79 | 26.32 | 26.32 | 31.58 | 31.58 | 42.86 | 47.62 | 52.38 | 52.38 | 57.14 | 57.14 | 52.38 | 71.43 |  |

Abbreviations: BASDAI = Bath Ankylosing Spondylitis Disease Activity Index; BASFI = Bath Ankylosing Spondylitis Functional Index; BASMI = Bath Ankylosing Spondylitis Metrology Index; CRP = C-Reactive Protein; SMD = Standardized Mean Difference.

ASAS20: Relative efficacy of intravenous golimumab vs infliximab for each scenario analysis, odds ratios and 95% confidence intervals

|  | Scenarios | | | | | | | | | | | | | | | | |
| --- | --- | --- | --- | --- | --- | --- | --- | --- | --- | --- | --- | --- | --- | --- | --- | --- | --- |
| Weeks of follow-up | 1 | 2 | 3 | 4 | 5 | 6 | 7 | 8 | 9 | 10 | 11 | 12 | 13 | 14 | 15 | 16 |  |
| 4 | 3.16  (0.64 to 15.56) | 4.2  (0.8 to 22.08) | 1.87  (0.34 to 10.37) | 2.67  (0.56 to 12.83) | 1.23  (0.26 to 5.78) | 1.91  (0.4 to 9.12) | 2.11  (0.45 to 9.88) | 1.08  (0.22 to 5.35) | 2.02  (0.41 to 9.88) | 1.55  (0.32 to 7.47) | 0.73  (0.16 to 3.34) | 0.62  (0.16 to 2.44) | 0.71  (0.15 to 3.43) | 0.6  (0.16 to 2.33) | 0.37  (0.09 to 1.49) | 0.76  (0.17 to 3.29) |  |
| 12 | 3.16  (0.61 to 16.29) | 3.47  (0.64 to 18.74) | 2.23  (0.38 to 13.02) | 3.16  (0.62 to 16) | 1.46  (0.3 to 7.1) | 2.01  (0.41 to 9.89) | 1.52  (0.31 to 7.54) | 1.04  (0.2 to 5.34) | 2.53  (0.5 to 12.87) | 1.3  (0.25 to 6.62) | 0.87  (0.19 to 4.12) | 0.98  (0.24 to 4.02) | 0.59  (0.11 to 3.07) | 0.93  (0.23 to 3.76) | 0.51  (0.12 to 2.21) | 0.57  (0.12 to 2.66) |  |
| 20 | 3.59  (0.66 to 19.46) | 3.23  (0.57 to 18.16) | 3.03  (0.49 to 18.87) | 5.06  (0.95 to 27.08) | 1.86  (0.37 to 9.35) | 5.46  (1.05 to 28.4) | 3.19  (0.62 to 16.44) | 2.07  (0.39 to 11.1) | 3.48  (0.66 to 18.36) | 1.68  (0.32 to 8.88) | 1.8  (0.37 to 8.74) | 1.37  (0.32 to 5.84) | 1.03  (0.19 to 5.58) | 1.52  (0.36 to 6.41) | 0.71  (0.16 to 3.21) | 0.68  (0.14 to 3.28) |  |
| 28 | **7.38**  **(1.35 to 40.3)** | **6.71**  **(1.18 to 38.3)** | 5.49  (0.87 to 34.59) | **7.04**  **(1.32 to 37.5)** | 4.73  (0.94 to 23.77) | **5.46**  **(1.08 to 27.51)** | **5.81**  **(1.15 to 29.4)** | 3.03  (0.58 to 15.71) | 3.94  (0.76 to 20.31) | 2.01  (0.39 to 10.25) | 1.59  (0.33 to 7.64) | 1.07  (0.25 to 4.5) | 0.94  (0.18 to 4.97) | 1.86  (0.45 to 7.76) | 0.56  (0.13 to 2.48) | 1.03  (0.22 to 4.84) |  |
| 36 | **9.05**  **(1.62 to 50.4)** | **8.2**  **(1.41 to 47.7)** | **10.06**  **(1.57 to 64.5)** | **7.21**  **(1.33 to 39.1)** | **5.62**  **(1.09 to 28.9)** | **6.67**  **(1.25 to 35.73)** | **5.27**  **(1.00 to 27.9)** | **7.9**  **(1.45 to 43.0)** | **7.31**  **(1.39 to 38.4)** | **6.57**  **(1.25 to 34.4)** | 3.09  (0.63 to 15.1) | 2.24  (0.52 to 9.57) | 1.02  (0.19 to 5.58) | 2.71  (0.64 to 11.47) | 1.17  (0.26 to 5.25) | 1.51  (0.31 to 7.23) |  |
| 44 | **9.05**  **(1.62 to 50.4)** | **10.21**  **(1.72 to 60.6)** | **8.01**  **(1.28 to 50.2)** | 5.00  (0.95 to 26.36) | **5.62**  **(1.09 to 28.9)** | **5.56**  **(1.06 to 29.33)** | **5.27**  **(1.00 to 27.9)** | **6.62**  **(1.23 to 35.6)** | **6.2**  **(1.19 to 32.2)** | **5.61**  **(1.08 to 29.1)** | 2.7  (0.56 to 13.09) | 1.96  (0.46 to 8.33) | 0.77  (0.14 to 4.15) | 2.37  (0.57 to 9.93) | 1.03  (0.23 to 4.57) | 1.32  (0.28 to 6.28) |  |
| 52 | 3.16  (0.61 to 16.29) | 3.36  (0.62 to 18.33) | 3.73  (0.63 to 22.04) | 2.33  (0.46 to 11.74) | 2.27  (0.46 to 11.1) | 2.95  (0.58 to 14.9) | 2.34  (0.47 to 11.73) | 3.11  (0.6 to 16.02) | 3.44  (0.68 to 17.34) | 3.18  (0.63 to 16.01) | 1.65  (0.35 to 7.81) | 1.22  (0.29 to 5.04) | 0.53  (0.10 to 2.8) | 1.45  (0.35 to 5.91) | 0.64  (0.15 to 2.77) | 0.82  (0.18 to 3.79) |  |

Note: Bold text denotes statistical significance (95% confidence interval excludes null value [1.0]). Odds ratio > 1 indicates favorability of golimumab IV compared to infliximab.

BASFI: *A priori* rankings of prognostic factors and treatment effect modifiers. The table below includes post-matching balance diagnostics under each scenario after implementing 1:1 nearest neighbor matching without replacement and a caliper of 0.2 standard deviations of the logit transform of the propensity score.

|  | **Scenarios** | | | | | | | | | | | | | | | | |
| --- | --- | --- | --- | --- | --- | --- | --- | --- | --- | --- | --- | --- | --- | --- | --- | --- | --- |
| **Factors ordered by *a priori* rankings** | **1** | **2** | **3** | **4** | **5** | **6** | **7** | **8** | **9** | **10** | **11** | **12** | **13** | **14** | **15** | **16** |  |
| BASFI | X | X | X | X | X | X | X | X | X | X | X | X | X | X | X | X |  |
| Age | X | X | X | X | X | X | X | X | X | X | X | X | X | X | X |  |  |
| Gender | X | X | X | X | X | X | X | X | X | X | X | X | X | X |  |  |  |
| Global assessment of disease activity | X | X | X | X | X | X | X | X | X | X | X | X | X |  |  |  |  |
| Total pain | X | X | X | X | X | X | X | X | X | X | X | X |  |  |  |  |  |
| BASDAI | X | X | X | X | X | X | X | X | X | X | X |  |  |  |  |  |  |
| SF-36 physical component score | X | X | X | X | X | X | X | X | X | X |  |  |  |  |  |  |  |
| BASMI | X | X | X | X | X | X | X | X | X |  |  |  |  |  |  |  |  |
| Human leukocyte antigen-B27 | X | X | X | X | X | X | X | X |  |  |  |  |  |  |  |  |  |
| Inflammation | X | X | X | X | X | X | X |  |  |  |  |  |  |  |  |  |  |
| Race | X | X | X | X | X | X |  |  |  |  |  |  |  |  |  |  |  |
| Region | X | X | X | X | X |  |  |  |  |  |  |  |  |  |  |  |  |
| CRP | X | X | X | X |  |  |  |  |  |  |  |  |  |  |  |  |  |
| Methotrexate use | X | X | X |  |  |  |  |  |  |  |  |  |  |  |  |  |  |
| SF-36 mental component score | X | X |  |  |  |  |  |  |  |  |  |  |  |  |  |  |  |
| Disease duration | X |  |  |  |  |  |  |  |  |  |  |  |  |  |  |  |  |
| Sample Size | 96 | 112 | 112 | 116 | 116 | 116 | 116 | 116 | 118 | 134 | 150 | 172 | 204 | 202 | 206 | 196 |  |
| Mean of SMDs | 0.08 | 0.09 | 0.1 | 0.12 | 0.13 | 0.15 | 0.16 | 0.17 | 0.15 | 0.17 | 0.27 | 0.25 | 0.28 | 0.3 | 0.29 | 0.28 |  |
| Proportion of SMDs ≥0.10 (%) | 41.18 | 35.29 | 44.44 | 47.06 | 52.94 | 38.89 | 52.63 | 57.89 | 42.11 | 47.37 | 78.95 | 73.68 | 68.42 | 78.95 | 68.42 | 78.95 |  |
| Proportion of SMDs ≥0.20 (%) | 5.88 | 5.88 | 5.56 | 5.88 | 17.65 | 22.22 | 26.32 | 36.84 | 21.05 | 21.05 | 57.89 | 52.63 | 52.63 | 63.16 | 57.89 | 52.63 |  |

Abbreviations: BASDAI = Bath Ankylosing Spondylitis Disease Activity Index; BASFI = Bath Ankylosing Spondylitis Functional Index; BASMI = Bath Ankylosing Spondylitis Metrology Index; CRP = C-Reactive Protein; SMD = Standardized Mean Difference.

BASFI: Relative efficacy of intravenous golimumab vs infliximab for each scenario analysis, mean differences and 95% confidence intervals

|  | Scenarios | | | | | | | | | | | | | | | | | |
| --- | --- | --- | --- | --- | --- | --- | --- | --- | --- | --- | --- | --- | --- | --- | --- | --- | --- | --- |
| Weeks of follow-up | | 1 | 2 | 3 | 4 | 5 | 6 | 7 | 8 | 9 | 10 | 11 | 12 | 13 | 14 | 15 | 16 |  |
| 4 | | -0.2  (-1.1 to 0.69) | 0.00  (-0.83 to 0.82) | -0.53  (-1.37 to 0.3) | -0.28  (-1.12 to 0.55) | -0.15  (-0.95 to 0.66) | -0.37  (-1.19 to 0.46) | -0.17  (-0.99 to 0.66) | -0.22  (-1.04 to 0.6) | 0.00  (-0.83 to 0.84) | 0.1  (-0.69 to 0.89) | 0.3  (-0.44 to 1.04) | 0.24  (-0.45 to 0.92) | 0.22  (-0.39 to 0.84) | 0.16  (-0.44 to 0.76) | 0.06  (-0.54 to 0.65) | 0.14  (-0.48 to 0.77) |  |
| 12 | | 0.03  (-0.86 to 0.93) | 0.46  (-0.37 to 1.28) | -0.08  (-0.92 to 0.76) | 0.01  (-0.82 to 0.84) | 0.2  (-0.61 to 1.00) | 0.13  (-0.7 to 0.95) | 0.28  (-0.54 to 1.11) | 0.06  (-0.76 to 0.89) | 0.3  (-0.54 to 1.14) | 0.52  (-0.27 to 1.32) | 0.61  (-0.13 to 1.36) | 0.5  (-0.18 to 1.19) | 0.58  (-0.03 to 1.2) | 0.46  (-0.14 to 1.06) | 0.32  (-0.28 to 0.91) | 0.4  (-0.22 to 1.02) |  |
| 20 | | -0.44  (-1.34 to 0.45) | -0.03  (-0.85 to 0.8) | -0.34  (-1.18 to 0.5) | -0.16  (-0.99 to 0.67) | -0.17  (-0.97 to 0.63) | -0.11  (-0.94 to 0.72) | -0.16  (-0.98 to 0.66) | -0.4  (-1.22 to 0.42) | -0.16  (-0.99 to 0.68) | 0.33  (-0.46 to 1.12) | 0.45  (-0.3 to 1.19) | 0.13  (-0.56 to 0.82) | 0.22  (-0.39 to 0.83) | 0.34  (-0.26 to 0.94) | 0.1  (-0.5 to 0.69) | 0.04  (-0.58 to 0.66) |  |
| 28 | | -0.23  (-1.12 to 0.67) | 0.13  (-0.69 to 0.96) | -0.37  (-1.21 to 0.47) | -0.26  (-1.1 to 0.57) | -0.08  (-0.88 to 0.72) | -0.1  (-0.92 to 0.73) | -0.13  (-0.95 to 0.7) | -0.27  (-1.09 to 0.55) | -0.21  (-1.05 to 0.63) | 0.4  (-0.39 to 1.19) | 0.57  (-0.18 to 1.31) | 0.25  (-0.44 to 0.93) | 0.3  (-0.31 to 0.91) | 0.52  (-0.08 to 1.13) | 0.19  (-0.4 to 0.78) | 0.23  (-0.39 to 0.85) |  |
| 36 | | -0.27  (-1.16 to 0.63) | -0.1  (-0.92 to 0.73) | -0.62  (-1.45 to 0.22) | -0.57  (-1.4 to 0.27) | -0.05  (-0.85 to 0.75) | -0.14  (-0.97 to 0.68) | -0.3  (-1.12 to 0.52) | -0.47  (-1.29 to 0.36) | -0.48  (-1.32 to 0.36) | 0.34  (-0.45 to 1.13) | 0.73  (-0.01 to 1.47) | 0.15  (-0.54 to 0.83) | 0.19  (-0.42 to 0.81) | 0.46  (-0.14 to 1.06) | 0.19  (-0.4 to 0.78) | 0.26  (-0.36 to 0.88) |  |
| 44 | | -0.39  (-1.29 to 0.5) | -0.2  (-1.02 to 0.63) | -0.79  (-1.63 to 0.05) | -0.71  (-1.55 to 0.12) | -0.27  (-1.07 to 0.54) | -0.42  (-1.25 to 0.41) | -0.39  (-1.21 to 0.44) | -0.58  (-1.4 to 0.25) | -0.56  (-1.4 to 0.28) | 0.19  (-0.6 to 0.98) | 0.63  (-0.11 to 1.38) | 0.01  (-0.67 to 0.7) | 0.06  (-0.55 to 0.68) | 0.33  (-0.27 to 0.93) | 0.06  (-0.53 to 0.66) | 0.12  (-0.51 to 0.74) |  |
| 52 | | -0.41  (-1.3 to 0.49) | -0.2  (-1.03 to 0.62) | -0.7  (-1.53 to 0.14) | -0.61  (-1.44 to 0.22) | -0.27  (-1.07 to 0.53) | -0.29  (-1.11 to 0.54) | -0.41  (-1.23 to 0.41) | -0.59  (-1.41 to 0.23) | -0.59  (-1.42 to 0.25) | 0.27  (-0.52 to 1.06) | 0.68  (-0.07 to 1.42) | 0.14  (-0.55 to 0.83) | 0.08  (-0.53 to 0.69) | 0.39  (-0.21 to 0.99) | 0.16  (-0.44 to 0.75) | 0.07 (-0.55 to 0.69) |  |

Note: Bold text denotes statistical significance (95% confidence interval excludes null value [0]). Mean difference < 0 indicates favorability of golimumab IV compared to infliximab.

CRP: *A priori* rankings of prognostic factors and treatment effect modifiers. The table below includes post-matching balance diagnostics under each scenario after implementing 1:1 nearest neighbor matching without replacement and a caliper of 0.2 standard deviations of the logit transform of the propensity score.

|  | **Scenarios** | | | | | | | | | |
| --- | --- | --- | --- | --- | --- | --- | --- | --- | --- | --- |
| **Factors ordered by *a priori* rankings** | **1** | **2** | **3** | **4** | **5** | **6** | **7** | **8** | **9** | **10** |
| Male | X | X | X | X | X | X | X | X | X | X |
| BASFI | X | X | X | X | X | X | X | X | X |  |
| CRP | X | X | X | X | X | X | X | X |  |  |
| Inflammation | X | X | X | X | X | X | X |  |  |  |
| Age | X | X | X | X | X | X |  |  |  |  |
| BASDAI | X | X | X | X | X |  |  |  |  |  |
| Global assessment of disease activity | X | X | X | X |  |  |  |  |  |  |
| Body mass index | X | X | X |  |  |  |  |  |  |  |
| Disease duration | X | X |  |  |  |  |  |  |  |  |
| Human leukocyte-B27 | X |  |  |  |  |  |  |  |  |  |
| Sample Size | 160 | 158 | 172 | 178 | 180 | 184 | 186 | 186 | 180 | 190 |
| Mean of SMDs | 0.05 | 0.06 | 0.14 | 0.12 | 0.12 | 0.18 | 0.17 | 0.15 | 0.17 | 0.2 |
| Proportion of SMDs ≥0.10 (%) | 25 | 16.67 | 66.67 | 25 | 33.33 | 58.33 | 58.33 | 58.33 | 41.67 | 66.67 |
| Proportion of SMDs ≥0.20 (%) | 0 | 0 | 16.67 | 16.67 | 16.67 | 41.67 | 50 | 33.33 | 41.67 | 41.67 |

Abbreviations: BASDAI = Bath Ankylosing Spondylitis Disease Activity Index; BASFI = Bath Ankylosing Spondylitis Functional Index; CRP = C-Reactive Protein; SMD = Standardized Mean Difference.

CRP: Relative efficacy of intravenous golimumab vs infliximab for each scenario analysis, mean differences and 95% confidence intervals

|  | **Scenarios** | | | | | | | | | |
| --- | --- | --- | --- | --- | --- | --- | --- | --- | --- | --- |
| Weeks of follow-up | **1** | **2** | **3** | **4** | **5** | **6** | **7** | **8** | **9** | **10** |
| 4 | -0.12  (-0.69 to 0.46) | -0.2  (-0.80 to 0.40) | -0.03  (-0.62 to 0.56) | -0.33  (-0.88 to 0.21) | -0.28  (-0.8 to 0.24) | -0.3  (-0.79 to 0.19) | -0.28  (-0.78 to 0.22) | -0.26  (-0.79 to 0.26) | 0.37  (-0.2 to 0.93) | 0.14  (-0.53 to 0.81) |
| 12 | 0.14  (-0.43 to 0.72) | 0.04  (-0.56 to 0.64) | 0.42  (-0.17 to 1.00) | 0.1  (-0.45 to 0.64) | 0.04  (-0.48 to 0.56) | 0.08  (-0.41 to 0.57) | 0.01  (-0.48 to 0.51) | 0.12  (-0.4 to 0.65) | 0.67  (0.11 to 1.23) | 0.51  (-0.16 to 1.18) |
| 20 | 0.3  (-0.27 to 0.88) | 0.15  (-0.46 to 0.75) | 0.52  (-0.07 to 1.11) | 0.16  (-0.39 to 0.71) | 0.22  (-0.3 to 0.73) | 0.19  (-0.29 to 0.68) | 0.12  (-0.38 to 0.62) | 0.24  (-0.29 to 0.76) | 0.7  (0.13 to 1.26) | 0.63  (-0.04 to 1.3) |
| 28 | 0.38  (-0.2 to 0.95) | 0.23  (-0.37 to 0.83) | 0.51  (-0.08 to 1.1) | 0.15  (-0.4 to 0.69) | 0.18  (-0.33 to 0.7) | 0.19  (-0.3 to 0.68) | 0.17  (-0.33 to 0.66) | 0.2  (-0.33 to 0.72) | 0.79  (0.22 to 1.35) | 0.66  (-0.01 to 1.33) |
| 36 | 0.24  (-0.34 to 0.81) | 0.16  (-0.44 to 0.76) | 0.48  (-0.1 to 1.07) | 0.08  (-0.47 to 0.62) | 0.15  (-0.36 to 0.67) | 0.01  (-0.48 to 0.5) | 0.07  (-0.43 to 0.57) | 0.16  (-0.37 to 0.68) | 0.57  (0.01 to 1.14) | 0.56  (-0.11 to 1.23) |
| 44 | 0.33  (-0.24 to 0.91) | 0.2  (-0.40 to 0.80) | 0.56  (-0.03 to 1.15) | 0.15  (-0.4 to 0.69) | 0.22  (-0.3 to 0.73) | 0.08  (-0.41 to 0.57) | 0.13  (-0.37 to 0.63) | 0.22  (-0.31 to 0.74) | 0.76  (0.20 to 1.33) | 0.62  (-0.05 to 1.29) |
| 52 | 0.3  (-0.27 to 0.87) | 0.25  (-0.35 to 0.85) | 0.44  (-0.14 to 1.03) | 0.05  (-0.49 to 0.6) | 0.09  (-0.43 to 0.61) | -0.06  (-0.55 to 0.43) | 0.03  (-0.47 to 0.52) | 0.02  (-0.51 to 0.54) | 0.77  (0.20 to 1.33) | 0.51  (-0.17 to 1.18) |

Note: Bold text denotes statistical significance (95% confidence interval excludes null value [0]). Mean difference < 0 indicates favorability of golimumab IV compared to infliximab.

**Appendix S3**

The following figure reports balance in the PS before and after nearest neighbor matching for (A) BASFI and (B) CRP. The matching algorithm was 1:1 nearest neighbor matching on all covariates (Scenarios 1 from Supplementary Appendix S1) with replacement and a caliper of 0.2 standard deviations of the logit transform of the propensity score.


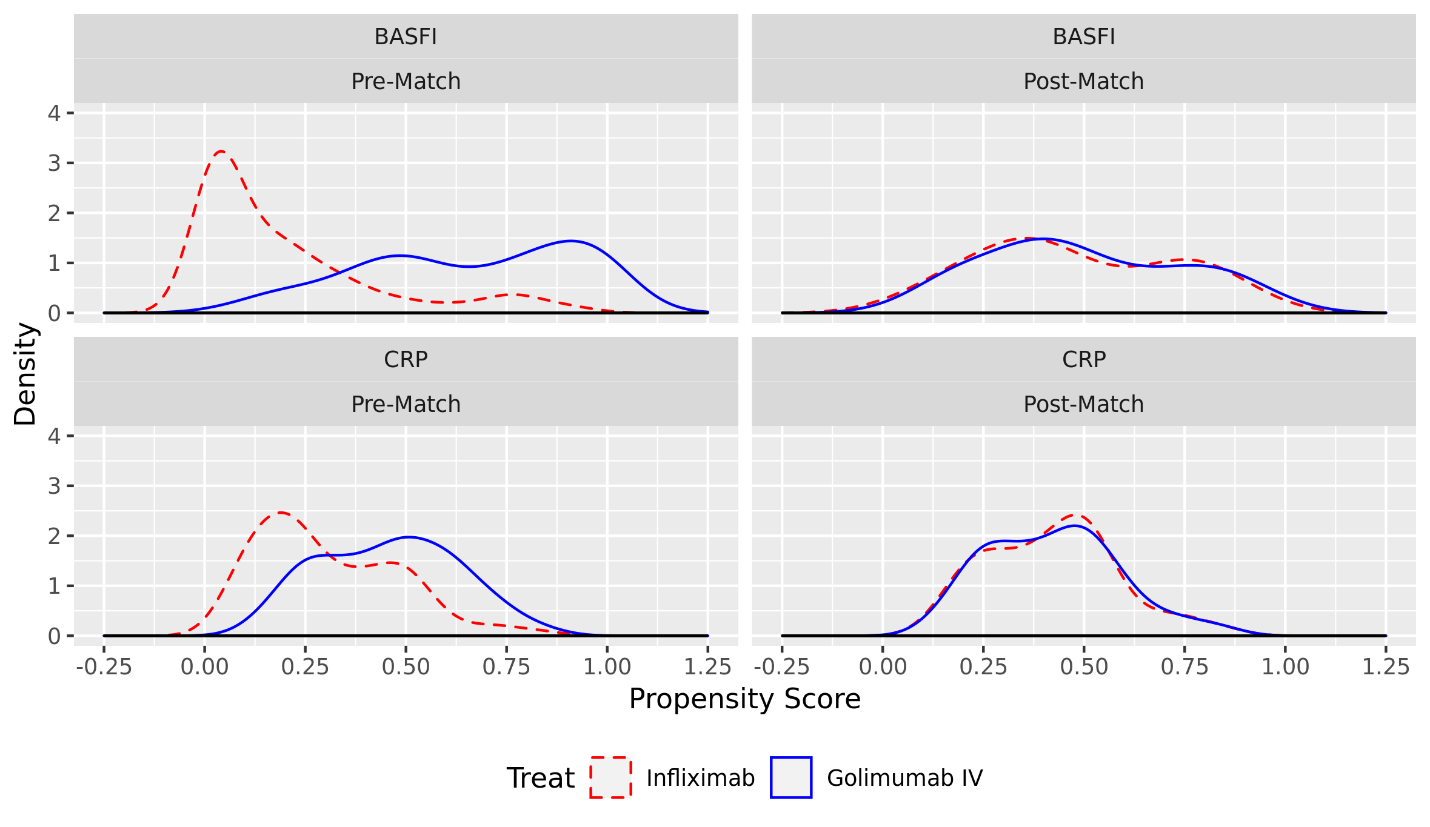


Abbreviations: BASFI = Bath Ankylosing Spondylitis Functional Index; CRP = C-Reactive Protein.

**Appendix S4**

Sensitivity analyses for ASAS20 using alternative propensity score matching and reweighting algorithms

|  | Odds Ratio (95% Confidence Interval), golimumab and infliximab | | | | | | | |
| --- | --- | --- | --- | --- | --- | --- | --- | --- |
|  | Complete Cases | | | | | | All Cases (Missing Values Imputed) | |
| Weeks of follow-up | 1:1 NN without replacement & Caliper = 0.2  **(PRIMARY)** | 1:1 NN without replacement & no caliper | 1:1 NN with replacement & no caliper | Optimal  Matching | 1:1 NN without replacement & Caliper = 0.25 | IPTW with ATT Weights | PRIMARY + LOCF | PRIMARY + NRI |
| 4 | 3.16 (0.64 to 15.56) | 1.44 (0.36 to 5.83) | 2.39 (0.42 to 13.58) | 1.44 (0.36 to 5.83) | 2.73 (0.57 to 13.12) | 1.65 (0.48 to 5.67) | 2.58 (0.48 to 13.72) | 2.52 (0.48 to 13.23) |
| 12 | 3.16 (0.61 to 16.29) | 1.52 (0.36 to 6.47) | 2.23 (0.38 to 13.17) | 1.52 (0.36 to 6.47) | 2.87 (0.59 to 14.11) | 1.19 (0.34 to 4.13) | 4.21 (0.76 to 23.21) | 4.08 (0.75 to 22.2) |
| 20 | 3.59 (0.66 to 19.46) | 2.13 (0.48 to 9.42) | 2.01 (0.32 to 12.58) | 2.13 (0.48 to 9.42) | 4.76 (0.92 to 24.59) | 2.8 (0.79 to 9.98) | **7.16 (1.24 to 41.22)** | **6.92 (1.22 to 39.36)** |
| 28 | **7.38 (1.35 to 40.34)** | 3.46 (0.8 to 14.93) | 3.5 (0.58 to 21.06) | 3.46 (0.8 to 14.93) | **7.61 (1.47 to 39.23)** | **5.74 (1.62 to 20.37)** | **12.13 (2.07 to 70.96)** | **11.73 (2.03 to 67.75)** |
| 36 | **9.05 (1.62 to 50.44)** | **3.99 (0.9 to 17.69)** | **8.55 (1.41 to 51.85)** | **3.99 (0.9 to 17.69)** | **7.87 (1.5 to 41.24)** | **11.24 (3.05 to 41.47)** | **9.93 (1.73 to 57.16)** | **9.6 (1.69 to 54.59)** |
| 44 | **9.05 (1.62 to 50.44)** | **3.49 (0.79 to 15.34)** | **7.49 (1.24 to 45.09)** | **3.49 (0.79 to 15.34)** | **9.65 (1.81 to 51.52)** | **9.54 (2.6 to 34.93)** | **9.93 (1.73 to 57.16)** | **9.6 (1.69 to 54.59)** |
| 52 | 3.16 (0.61 to 16.29) | 2.15 (0.5 to 9.19) | 4.62 (0.78 to 27.25) | 2.15 (0.5 to 9.19) | 3.34 (0.68 to 16.51) | **5.8 (1.63 to 20.68)** | 4.25 (0.78 to 23.25) | 4.12 (0.76 to 22.24) |
| Sample Size | 118 | 184 | 123 | 184 | 120 | 300 | 124 | 124 |
| **Golimumab IV** | 59 | 92 | 92 | 92 | 60 | 100 | 62 | 62 |
| **Infliximab** | 59 | 92 | 41 | 92 | 60 | 200 | 62 | 62 |
| Mean SMDs | 0.07 | 0.18 | 0.14 | 0.18 | 0.05 | 0.20 | 0.06 | 0.06 |
| Proportion of SMDs ≥0.10 (%) | 26.32 | 76.19 | 47.62 | 76.19 | 10.53 | 66.67 | 26.32 | 26.32 |
| Proportion of SMDs ≥0.20 (%) | 0.00 | 33.33 | 28.57 | 33.33 | 0.00 | 38.1 | 5.26 | 5.26 |

Abbreviatons: ATT = Average Treatment Effect on the Treated; IPTW = inverse probability of treatment weighting; NN = nearest neighbor; LOCF = last observation carried forward; NRI = non-responder imputation; SMD = standardized mean difference

Notes: Bold text denotes statistical significance (95% confidence interval excludes null value [1.0]). Odds ratio >1 indicates favorability of golimumab IV compared to infliximab.

Sensitivity analyses for BASFI using alternative propensity score matching and reweighting algorithms

|  | Mean Difference (95% Confidence Interval), golimumab and infliximab | | | | | | |
| --- | --- | --- | --- | --- | --- | --- | --- |
|  | Complete Cases | | | | | | All Cases (Missing Values Imputed) |
| Weeks of follow-up | 1:1 NN without replacement & Caliper = 0.2  **(PRIMARY)** | 1:1 NN without replacement & no caliper | 1:1 NN with replacement & no caliper | Optimal  Matching | 1:1 NN without replacement & Caliper = 0.25 | IPTW with ATT Weights | PRIMARY + LOCF |
| 4 | -0.2 (-1.1 to 0.69) | -0.05 (-0.67 to 0.57) | -0.06 (-0.94 to 0.83) | -0.05 (-0.67 to 0.57) | -0.61 (-1.54 to 0.33) | -0.35 (-0.86 to 0.17) | -0.41 (-1.28 to 0.45) |
| 12 | 0.03 (-0.86 to 0.93) | 0.18 (-0.44 to 0.8) | 0.1 (-0.79 to 0.98) | 0.18 (-0.44 to 0.8) | -0.14 (-1.07 to 0.8) | -0.06 (-0.58 to 0.46) | -0.24 (-1.1 to 0.63) |
| 20 | -0.44 (-1.34 to 0.45) | -0.16 (-0.78 to 0.45) | -0.4 (-1.29 to 0.49) | -0.16 (-0.78 to 0.45) | -0.53 (-1.46 to 0.4) | -0.39 (-0.91 to 0.13) | -0.65 (-1.51 to 0.22) |
| 28 | -0.23 (-1.12 to 0.67) | -0.1 (-0.71 to 0.52) | -0.27 (-1.15 to 0.62) | -0.1 (-0.71 to 0.52) | -0.41 (-1.34 to 0.53) | -0.47 (-0.99 to 0.04) | -0.61 (-1.48 to 0.25) |
| 36 | -0.27 (-1.16 to 0.63) | -0.24 (-0.86 to 0.38) | -0.7 (-1.58 to 0.19) | -0.24 (-0.86 to 0.38) | -0.58 (-1.51 to 0.36) | -0.54 (-1.06 to -0.02) | -0.51 (-1.37 to 0.36) |
| 44 | -0.39 (-1.29 to 0.5) | -0.37 (-0.98 to 0.25) | -0.82 (-1.71 to 0.07) | -0.37 (-0.98 to 0.25) | -0.72 (-1.65 to 0.21) | -0.66 (-1.18 to -0.15) | -0.67 (-1.54 to 0.19) |
| 52 | -0.41 (-1.3 to 0.49) | -0.29 (-0.91 to 0.33) | -0.62 (-1.51 to 0.26) | -0.29 (-0.91 to 0.33) | -0.61 (-1.55 to 0.32) | -0.33 (-0.85 to 0.19) | -0.65 (-1.52 to 0.21) |
| Sample Size | 96 | 200 | 132 | 200 | 96 | 300 | 106 |
| **Golimumab IV** | 48 | 100 | 100 | 100 | 48 | 100 | 53 |
| **Infliximab** | 48 | 100 | 32 | 100 | 48 | 200 | 53 |
| Mean SMDs | 0.08 | 0.22 | 0.17 | 0.22 | 0.1 | 0.23 | 0.06 |
| Proportion of SMDs ≥0.10 (%) | 41.18 | 73.68 | 42.11 | 73.68 | 44.44 | 73.68 | 23.53 |
| Proportion of SMDs ≥0.20 (%) | 5.88 | 47.37 | 36.84 | 47.37 | 16.67 | 47.37 | 0 |

Abbreviations: ATT = Average Treatment Effect on the Treated; IPTW = inverse probability of treatment weighting; NN = nearest neighbor; LOCF = last observation carried forward; SMD = standardized mean difference

Notes: Bold text denotes statistical significance (95% confidence interval excludes null value [0]). Mean difference <0 indicates favorability of golimumab IV compared to infliximab.

Sensitivity analyses for CRP using alternative propensity score matching and reweighting algorithms

|  | Mean Difference (95% Confidence Interval), golimumab and infliximab | | | | | | |
| --- | --- | --- | --- | --- | --- | --- | --- |
|  | Complete Cases | | | | | | All Cases (Missing Values Imputed) |
| Weeks of follow-up | 1:1 NN without replacement & Caliper = 0.2  **(PRIMARY)** | 1:1 NN without replacement & no caliper | 1:1 NN with replacement & no caliper | Optimal  Matching | 1:1 NN without replacement & Caliper = 0.25 | IPTW with ATT Weights | PRIMARY + LOCF |
| 4 | -0.12 (-0.69 to 0.46) | -0.32 (-0.84 to 0.19) | -0.14 (-0.74 to 0.47) | -0.34 (-0.86 to 0.17) | -0.17 (-0.77 to 0.42) | 0.17 (-0.4 to 0.75) | -0.25 (-0.81 to 0.31) |
| 12 | 0.14 (-0.43 to 0.72) | 0.05 (-0.47 to 0.56) | 0.25 (-0.35 to 0.85) | 0.02 (-0.5 to 0.53) | 0.13 (-0.46 to 0.72) | 0.44 (-0.14 to 1.01) | 0.11 (-0.45 to 0.67) |
| 20 | 0.3 (-0.27 to 0.88) | 0.15 (-0.37 to 0.67) | 0.36 (-0.24 to 0.96) | 0.13 (-0.39 to 0.64) | 0.11 (-0.48 to 0.7) | 0.47 (-0.11 to 1.05) | 0.18 (-0.38 to 0.74) |
| 28 | 0.38 (-0.2 to 0.95) | 0.17 (-0.35 to 0.68) | 0.41 (-0.19 to 1.01) | 0.15 (-0.37 to 0.66) | 0.16 (-0.44 to 0.75) | 0.38 (-0.2 to 0.96) | 0.12 (-0.44 to 0.68) |
| 36 | 0.24 (-0.34 to 0.81) | 0.04 (-0.47 to 0.56) | 0.29 (-0.31 to 0.89) | 0.02 (-0.49 to 0.54) | 0.06 (-0.53 to 0.65) | 0.3 (-0.28 to 0.88) | 0.05 (-0.51 to 0.61) |
| 44 | 0.33 (-0.24 to 0.91) | 0.12 (-0.39 to 0.64) | 0.38 (-0.23 to 0.98) | 0.1 (-0.41 to 0.62) | 0.11 (-0.48 to 0.71) | 0.38 (-0.2 to 0.96) | 0.12 (-0.44 to 0.68) |
| 52 | 0.3 (-0.27 to 0.87) | 0.03 (-0.48 to 0.55) | 0.19 (-0.41 to 0.79) | 0.01 (-0.5 to 0.53) | 0.11 (-0.48 to 0.7) | 0.23 (-0.35 to 0.81) | -0.02 (-0.58 to 0.54) |
| Sample Size | 160 | 188 | 156 | 188 | 164 | 305 | 184 |
| **Golimumab IV** | 80 | 94 | 94 | 94 | 82 | 104 | 92 |
| **Infliximab** | 80 | 94 | 62 | 94 | 82 | 201 | 92 |
| Mean SMDs | 0.05 | 0.08 | 0.08 | 0.07 | 0.04 | 0.13 | 0.04 |
| Proportion of SMDs ≥0.10 (%) | 25.00 | 41.67 | 25 | 33.33 | 0.00 | 33.33 | 0.00 |
| Proportion of SMDs ≥0.20 (%) | 0.00 | 0.00 | 0.00 | 0.00 | 0.00 | 25 | 0.00 |

Abbreviations: ATT = Average Treatment Effect on the Treated; IPTW = inverse probability of treatment weighting; NN = nearest neighbor; LOCF = last observation carried forward; SMD = standardized mean difference

Notes: Bold text denotes statistical significance (95% confidence interval excludes null value [0]). Mean difference <0 indicates favorability of golimumab IV compared to infliximab.

**Appendix S5** Sensitivity analyses comparing propensity score matching and multivariable regression adjustment

|  | ASAS20 |  | BASFI |  | CRP |  |
| --- | --- | --- | --- | --- | --- | --- |
|  | Odds Ratio (95% Confidence Interval*) | | Mean Difference (95% Confidence Interval*) | | Mean Difference (95% Confidence Interval*) | |
| Weeks of follow-up | 1:1 NN without replacement & Caliper = 0.2 | Bayesian Multivariable Regression | 1:1 NN without replacement & Caliper = 0.2 | Bayesian Multivariable Regression | 1:1 NN without replacement & Caliper = 0.2 | Bayesian Multivariable Regression |
|  | **(PRIMARY)** |  | **(PRIMARY)** |  | **(PRIMARY)** |  |
| 4 | 3.16 (0.64 to 15.56) | 2.33 (0.50 to 10.46) | -0.2 (-1.1 to 0.69) | -0.11 (-0.67 to 0.42) | -0.12 (-0.69 to 0.46) | -0.21 (-0.47 to 0.05) |
| 12 | 3.16 (0.61 to 16.29) | 2.62 (0.56 to 12.26) | 0.03 (-0.86 to 0.93) | 0.05 (-0.51 to 0.60) | 0.14 (-0.43 to 0.72) | 0.10 (-0.16 to 0.36) |
| 20 | 3.59 (0.66 to 19.46) | 4.08 (0.82 to 20.44) | -0.44 (-1.34 to 0.45) | -0.27 (-0.84 to 0.29) | 0.3 (-0.27 to 0.88) | **0.27 (0.01 to 0.53)** |
| 28 | **7.38 (1.35 to 40.34)** | 3.94 (0.84 to 18.80) | -0.23 (-1.12 to 0.67) | -0.20 (-0.77 to 0.34) | 0.38 (-0.2 to 0.95) | **0.27 (0.02 to 0.55)** |
| 36 | **9.05 (1.62 to 50.44)** | **6.30 (1.27 to 30.81)** | -0.27 (-1.16 to 0.63) | -0.22 (-0.77 to 0.34) | 0.24 (-0.34 to 0.81) | 0.17 (-0.09 to 0.43) |
| 44 | **9.05 (1.62 to 50.44)** | **5.57 (1.09 to 27.47)** | -0.39 (-1.29 to 0.5) | -0.34 (-0.89 to 0.19) | 0.33 (-0.24 to 0.91) | 0.26 (-0.003 to 0.52) |
| 52 | 3.16 (0.61 to 16.29) | 3.29 (0.69 to 15.42) | -0.41 (-1.3 to 0.49) | -0.40 (-0.96 to 0.13) | 0.3 (-0.27 to 0.87) | 0.14 (-0.12 to 0.41) |
| Sample Size | 118 | 266 | 96 | 273 | 160 | 261 |
| **Golimumab IV** | 59 | 92 | 48 | 173 | 80 | 167 |
| **Infliximab** | 59 | 174 | 48 | 100 | 80 | 91 |
| Mean SMDs | 0.07 | NR | 0.08 | NR | 0.05 | NR |
| Proportion of SMDs ≥0.10 (%) | 26.32 | NR | 41.18 | NR | 25 | NR |
| Proportion of SMDs ≥0.20 (%) | 0 | NR | 5.88 | NR | 0 | NR |

Abbreviations: BASFI = Bath Ankylosing Spondylitis Functional Index; CRP = C-Reactive Protein; IV = intravenous; NN = nearest neighbor; SMD = standardized mean difference.

Notes: Bold text denotes statistical significance (95% confidence interval excludes null value [0]). Odds ratio >1 and mean difference <0 indicates favorability of golimumab compared to infliximab.

* Bayesian methods were used for all multivariable regression models.95% credible intervals were used to represent uncertainty of effect estimates obtained via Bayesian multivariable regression models. Given a model (prior distribution) and dataset, the 95% credible interval contains 95% of posterior distribution of effect estimate (either odds ratio or mean difference), from the 2.5^th^ to 97.5^th^ percentiles of the posterior distribution.
