# Supplementary figures and images for: Propensity score matching/reweighting analysis comparing intravenous golimumab to infliximab for ankylosing spondylitis using data from the GO-ALIVE and ASSERT trials
Source: Clin Rheumatol. 2020 May 4;39(10):2907–17. doi: 10.1007/s10067-020-05051-1 (PMC7497341; doi:10.1007/s10067-020-05051-1)

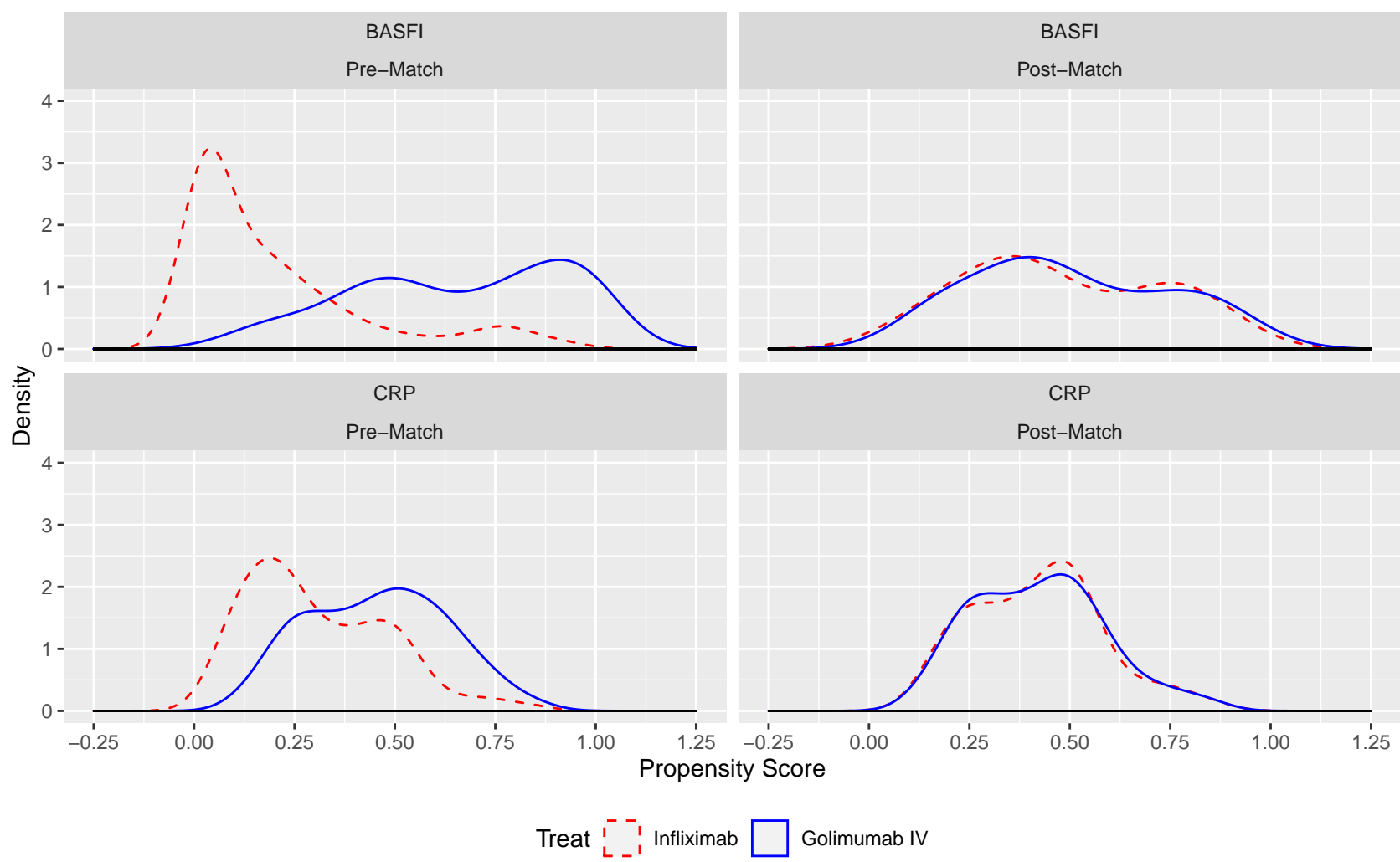

Supplement: Supplementary file 2 — (PDF 32.8 kb) [file 10067_2020_5051_MOESM2_ESM.pdf]
